# Efficient All-Polymer Solar Cells with Sequentially Processed Active Layers

**DOI:** 10.3390/polym14102058

**Published:** 2022-05-18

**Authors:** Chaoyue Zhao, Hui Huang, Lihong Wang, Guoping Zhang, Guanyu Lu, Han Yu, Guanghao Lu, Yulai Han, Mingxia Qiu, Shunpu Li, Guangye Zhang

**Affiliations:** 1 College of New Materials and New Energies, Shenzhen Technology University, Shenzhen 518118, China; zhaochaoyue2020@email.szu.edu.cn (C.Z.); huanghui@sztu.edu.cn (H.H.); wanglihong@sztu.edu.cn (L.W.); 202003020103@stumail.sztu.edu.cn (G.Z.); hanyulai@sztu.edu.cn (Y.H.); qiumingxia@sztu.edu.cn (M.Q.); lishunpu@sztu.edu.cn (S.L.); 2 Frontier Institute of Science and Technology, Xi’an Jiaotong University, Xi’an 710054, China; luguanyu@stu.xjtu.edu.cn (G.L.); guanghao.lu@mail.xjtu.edu.cn (G.L.); 3 Department of Chemistry and Hong Kong Branch of Chinese National Engineering Research Center for Tissue Restoration and Reconstruction, The Hong Kong University of Science and Technology, Clear Water Bay, Hong Kong 999077, China; hyuak@connect.ust.hk

**Keywords:** all-polymer solar cells, sequential processing, polymerized small-molecule acceptors

## Abstract

In this work, we apply the sequential processing (SqP) method to address the relatively low electron mobility in recent all-polymer solar cells (all-PSCs) based on the polymerized small-molecule acceptor (PSMA). Compared to the blend-casting (BC) method, all-PSCs composed of PM6/PY-IT via the SqP method show boosted electron mobility and a more balanced charge carrier transport, which increases the FF of the SqP device and compensates for the short-circuit current loss, rendering comparable overall performance with the BC device. Through film-depth-dependent light absorption spectroscopy, we analyze the sub-layer absorption and exciton generation rate in the vertical direction of the device, and discuss the effect of the increased electron mobility on device performance, accordingly.

## 1. Introduction

As a promising green and renewable energy technology, thin-film organic solar cells (OSCs) have received worldwide interest and have developed quickly in recent decades due to their advantages, such as light weight, low cost, semitransparency, and flexibility [1,2,3,4,5,6,7]. Non-fullerene OSCs, in which small-molecule acceptors (SMAs) or polymer acceptors replace fullerene derivatives, are attracting significant attention in light of their easily tunable energetics and good light absorption and large charge mobility. The power conversion efficiencies (PCEs) of non-fullerene OSCs approaching 19% have been reported [8].

With the synthesis of new materials and the exploration of new structures, all-polymer solar cells (all-PSCs) consisting of p-type polymer donors and n-type polymer acceptors have attracted increased attention due to some of their distinctive features, such as enhanced electronic structure tunability, outstanding mechanical durability and stability, and excellent film-formation capability suitable for roll-to-roll manufacturing [9,10,11]. Notably, polymerizing small--molecule acceptors (SMAs) to construct polymer acceptors (PSMAs) has boosted the PCEs of the all-PSCs to over 15% recently [12,13,14,15,16,17], yet this still lags behind the state-of-the-art efficiencies from non-fullerene OSCs [18]. This is mainly due to the difficulty in morphology regulation of bulk-heterojunction (BHJ) blend films in all-PSC active layers [19]. The active layer fabricated by the polymeric donor and acceptor usually affords an amorphous BHJ morphology with low polymer crystallinity and pure phase when the using one step blend-casting (BC) method [20]. This is mainly because polymers have a strong tendency to intertwine and entangle with neighboring chains, even in the solution [21].

To optimize the BHJ morphology, sequential processing (SqP), also called layer-by--layer deposition, has been considered as an effective way to form a pseudo-bilayer configuration via two-step deposition of the donor and acceptor, sequentially, which is beneficial to enhance vertical phase separation and afford a purer donor/acceptor phase to accelerate the hole/electron transport [22,23,24,25,26]. Some literature has confirmed that using the SqP method can fabricate higher efficiency of fullerene OSCs/non-fullerene small-molecule acceptor OSCs [27,28,29,30]. In addition, as donor and acceptor layers can be processed and optimized independently, the SqP method makes the active layer less dependent on the donor/acceptor ratio, solvent additive, etc., compared with the BC method. Therefore, it is easier to enhance film quality of active layers by choosing the SqP approach over BC, indicating that SqP can provide higher performance with better reproducibility in larger-area devices than BC [25]. Zhang et al., in 2018, reported high-performance large-area OSCs fabricated by using the SqP technique [31]. Despite these advantages, the SqP method is barely used for the advancement of all-PSCs, especially those based on PSMAs, let alone high efficiencies. This is because the formation of a SqP active layer relies on the swelling of the underlayer by the top solution and the interdiffusion between the two donor and acceptor materials, and the polymers’ high molecular weights hinder themselves when diffusing into the donor film in all-PSCs.

**Figure 1:**
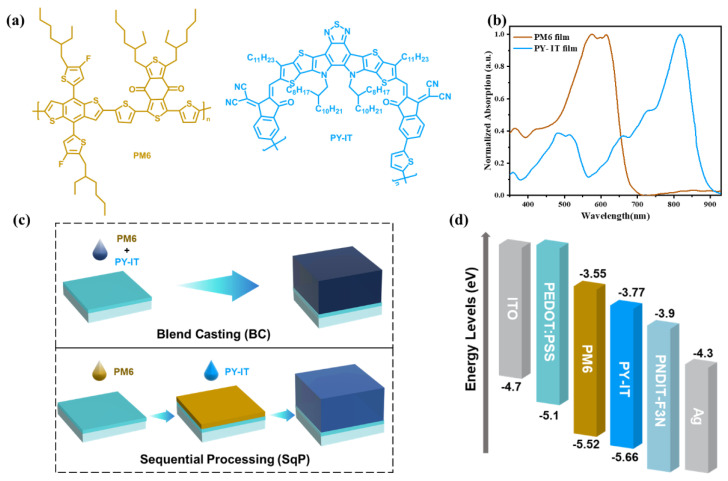
(**a**) Chemical structures of the materials. (**b**) UV-Vis absorption spectra of thin films. (**c**) Schematic diagram of blend-casting and sequential processing. (**d**) Energy level diagram of polymers. The bottom and top of the bar represent HOMO and LUMO, respectively.

In this work, we present a sequentially processed all-PSC with PM6 as the electron donor and PY-IT, a PSMA, as the electron acceptor (Figure 1a). The nanomorphology of PM6 and PY-IT is finely manipulated through SqP assisted by a solvent additive and thermal annealing, but the same selective organic solvent used in all-PSCs devices by either SqP or BC. A small amount of 1-chloronaphthalene (CN) as a solvent additive was added during the deposition of the top solution to tune the swelling of the underlayer. As a result, optimal PCEs of 15% were successfully achieved for all-PSCs composed of PM6/PY-IT by using the SqP method. Furthermore, to study the effect of swelling, we prepare another device whose underlayer (PM6) was thermally annealed before the deposition of the top solution, which is denoted by “donor thermally annealed (DTA)”. These SqP and BC devices are characterized by film-depth-dependent absorption spectroscopy (FLAS), FTPS-EQE, ideality factor analysis, etc. It reveals that the SqP method makes PM6/PY-IT have a larger fill factor (FF) due to the more balanced carrier mobility and less bimolecular recombination. Furthermore, analysis on the FLAS result shows that there are more excitons generated in the bottom half of the device where the electrons need to transport through a longer distance to reach the top (cathode) compared to the holes to the bottom, which further highlights the significance of increasing the electron mobility via the SqP method.

## 2. Materials and Methods

### 2.1. Materials

Poly[(2,6-(4,8-bis(5-(2-ethylhexyl-3-fluoro)thiophen-2-yl)-benzo[1,2b:4,5-b′]dithiophene))-alt-(5,5-(1′,3′-di-2-thienyl-5′,7′-bis(2-ethylhexyl)benzo[1′,2′-c:4′,5′c′]dithiophene-4,8-dione)] (PM6) was purchased from Solarmer Material Inc. (Beijing, China) poly[(2,2′-((2Z,2′Z)-((12,13-bis(2-octyldodecyl)-3,9-diundecyl-12,13-dihydro[1,2,5]thiadiazolo[3,4e]thieno[2″,3″:4′,5′]thieno[2′,3′:4,5]pyrrolo [3,2-g]thieno[2′,3′:4,5]thieno[3,2-b]-indole-2,10-diyl)bis(methanylylidene))bis(5-methyl-3-oxo-2,3-dihydro-1H-indene-2,1-diylidene)) dimalononitrile-co-2,5-thiophene (PY-IT) and poly[(9,9-bis(3′-(N,N-dimethylamino)propyl)2,7-fluorene) -alt-5,5′-bis(2,2′-thiophene) -2,6-naphthalene1,4,5,8-tetracaboxylic-N,N′-di(2-ethylhexyl)imide] (PNDIT-F3N) were purchased from eFlexPV Limited (Guangzhou, China). Poly(3,4-ethylenedioxythiophene) polystyrene sulfonate (PEDOT:PSS) (Clevios P VP 4083) was purchased from Heraeus Inc., Hanau, Germany. All the other reagents and chemicals were purchased from Sigma-Aldrich or Aladdin and used as received.

### 2.2. Experimental Equipment and Facilities

**Device fabrication.** Solar cells were fabricated in a conventional device configuration of ITO/PEDOT:PSS/active layers/PNDIT-F3N/Ag. The ITO substrates were scrubbed by detergent and then sonicated with deionized water, acetone, and isopropanol, subsequently, and dried overnight in an oven. The glass substrates were treated with UV-Ozone for 30 min before use. PEDOT:PSS was spin-casted onto the ITO substrates at 5000 rpm for 30 s, and then dried at 120 °C for 10 min in air.

The different kinds of devices used were as follows:

(1) For PM6:PY-IT (BC), the PM6:PY-IT blends (1:1.2 weight ratio) were dissolved in chloroform (the concentration of the donor was 7 mg mL^−1^ for all blends) or toluene (the concentration of the donor was 7 mg mL^−1^ for all blends), with 1-chloronaphthalene (1% vol) as an additive, and stirred overnight in a nitrogen-filled glove box. The chloroform blend solution was spin-casted at 2500 rpm for 30 s onto the PEDOT:PSS films, followed by a temperature annealing of 95 °C for 5 min. The 95 °C toluene blend solution was spin-casted at 2500 rpm for 30 s onto the PEDOT:PSS films, followed by a temperature annealing of 95 °C for 5 min.

(2) For PM6/PY-IT (SqP), the PM6 was dissolved in chloroform (the concentration of the donor was 8 mg mL^−1^), and the acceptor PY-IT was also dissolved in chloroform (the concentration of the donor was 9 mg mL^−1^) and 1-chloronaphthalene (2% vol) as an additive, and both solutions were stirred overnight in a nitrogen-filled glove box. The donor solution was spin-casted at 2500 rpm for 30 s onto the PEDOT:PSS films, and then the donor solution was spin-casted at 3000 rpm for 30 s onto the donor films, followed by a temperature annealing of 95 °C for 5 min.

(3) For PM6/PY-IT (SqP-DTA), the thermal annealing at 90 °C for 5 min was carried out after the donor film was spin-casted, then the other steps were the same as outlined in (2).

For all types of devices, methanol with a 0.5% vol. acetic acid blend solution of PNDIT-F3N at a concentration of 0.5 mg mL^−1^ was spin-coated onto the active layer at 2000 rpm for 30 s. Around 100 nm of Ag was evaporated under 1 × 10^−4^ Pa through a shadow mask, then the encapsulation was carried out.

**Device characterization.** The current density–voltage (*J*-*V*) curves of all encapsulated devices were measured using a Keithley 2400 Source Meter ()in air under AM 1.5G (100 mW cm^−2^) using a Newport solar simulator (Taiwan, China). The light intensity was calibrated using a standard Si diode (with KG5 filter, purchased from PV Measurement to bring spectral mismatch to unity. An optical microscope (Olympus BX51 was used to define the device area (8.5 mm^2^). EQEs were measured using an Enlitech QE-S EQE system (Taiwan, China) equipped with a standard Si diode. Monochromatic light was generated from a Newport 300 W lamp source 

### 2.3. Analysis and Characterization

**SCLC Measurements:** The electron and hole mobilities were measured using the space-charge limited current (SCLC) method. The device architecture of the electron-only devices was ITO/ZnO/active layer/Ca/Ag, and that of the hole-only devices was ITO/MoO_x_/active layer/MoO_x_/Ag. The charge carrier mobilities were determined by fitting the dark current into the model of a single carrier SCLC according to the equation: *J* = 9*ε*_0_*ε*_r_*μV*^2^/8*d*^3^, where *J* is the current density, *d* is the film thickness of the active layer, *μ* is the charge carrier mobility, *ε*_r_ is the relative dielectric constant of the transport medium, and *ε*_0_ is the permittivity of free space. *V* = *V*_app_ − *V*_bi_, where *V*_app_ is the applied voltage and *V*_bi_ is the offset voltage. The carrier mobilities were calculated from the slope of the *J*~*V*^2^ curves.

**FTPS**-**EQE:** FTPS measurements were conducted on the same devices used in *J*-*V* measurements using an integrated system (PECT-600) purchased from Enli Technology Co., Ltd. (Taiwan, China). The range of measurements was 500–1800 nm. The signal due to noise was cut out for all devices below 1.1–1.3 eV.

**Film**-**depth**-**dependent light absorption and composition distribution:** Film-depth-dependent light absorption spectra were acquired by an in situ spectrometer (PU100, Shaanxi Puguang Weishi Co. Ltd.) (Shaanxi, China) equipped with a soft plasma-ion source. The power-supply for generating the soft ionic source was 100 W, with input oxygen pressure ~ 10 Pa. The film surface is incrementally etched by the soft ion source, without damage to the materials underneath the surface, which is in situ monitored by a spectrometer. From the evolution of the spectra and the Beer-Lambert’s Law, film-depth-dependent absorption spectra were extracted.

The composition distribution along the film-depth direction was obtained from the film-depth-dependent spectra. The exciton generation contour was numerically simulated upon inputting film-depth-dependent light absorption spectra into a modified optical transfer-matrix approach. The detailed experimental and numerical methods are available elsewhere [31,32].

## 3. Results and Discussion

The chemical structural formulas of PM6 and PY-IT are shown in Figure 1a. The photovoltaic properties of these three systems were investigated in a device structure consisting of indium tin oxide (ITO)/poly(3,4-ethylene dioxythiophene):polystyrene sulfonate (PEDOT:PSS)/(active layer with BC approach or SqP approach)/PNDIT-F3N/Ag. The complete fabrication of the solution-processed BC and SqP, and the thickness control of each layer, are provided in the Materials and Experiment Section. As shown in Figure 1b, the polymer donor PM6 exhibited strong absorption from 300 to 700 nm. The absorption spectrum of neat PY-IT in the range of 700−900 nm was complementary to that of PM6, in favor of enhancing the photocurrent. The two types of blend films showed nearly identical absorption profiles, with strong and wide absorption within 300−900 nm as a result of the absorption superposition of the two components.

Figure 1c depicts the device structure fabricated by two different processing approaches. As shown in the upper part of Figure 1c, it was obtained by one-step blend-casting (BC) from the donor PM6 and acceptor PY-IT mixed solution. The sequential processing (SqP) with the underlayer donor PM6 and the upper acceptor PY-IT, being sequentially spin-casted, is presented in the lower part of Figure 1c. The highest occupied molecular orbital (HOMO) and lowest unoccupied molecular orbital (LUMO) energy levels of the active-layer materials (Figure 1d) were taken from the literature [33,34].

**Figure 2:**
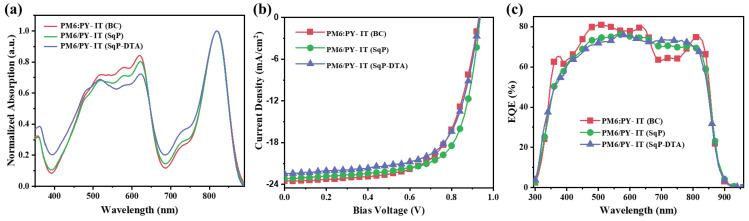
(**a**) UV-Vis absorption spectra of thin films (normalized to acceptor absorption peaks) based on BC, SqP, or SqP-DTA systems. (**b**) Current density-voltage *(J-V)* curves, and (**c**) corresponding external quantum efficiency (EQE) spectra of PM6/PY-IT-based devices.

Figure 2a presents the absorption spectra of all-PSCs thin films based on BC, SqP, and SqP (DTA) systems. The corresponding specific device performance parameters are listed in Table 1. With some variations in the spectral range of 500–630 nm, the overall shape and intensity of the three blends were similar. The lower absorption for the SqP-DTA film in this region suggests that the extra annealing on the underlayer reduces the swelling of it during the deposition of the acceptor, so that the ratio of donor to acceptor for this DTA film was relatively lower.

Figure 2b exhibits *J*-*V* characteristic curves of all-PSCs based on BC, SqP, and SqP (DTA) systems. The corresponding specific device performance parameters are listed in Table 1. The PM6:PY-IT (BC) showed a maximum PCE of 15.15%, with a short-circuit current density (*J*_SC_) of 23.70 mA·cm^−2^, a *V*_OC_ of 0.937 V, and a fill factor (FF) of 67.7%. Considering those values of PM6:PY-IT (BC) as a benchmark, the PM6/PY-IT (SqP) exhibited a slightly lower *J*_SC_ of 23.19 mA·cm^−2^, a nearly unchanged *V*_OC_ (0.936 V), and a higher FF of 69.1%, and the PM6/PY-IT (SqP-DTA) showed a significantly lower *J*_SC_ of 22.36 mA·cm^−2^, a more reduced FF (65.7%), and a decreased *V*_OC_ (0.932 V), which led to the lowest PCE of 13.69% among the three. The result indicates that donor thermally annealed (DTA) is not ideal to tune BHJ morphology by SqP. The *J*_SC_ differences for those film devices with the BC and SqP methods were also cross-checked with external quantum efficiency (EQE) measurements (see Figure 2c). The results showed that the PM6:PY-IT (BC) system had a slightly higher EQE value, in the range of 500–630 nm, than that of the PM6/PY-IT (SqP) system. The calculated *J*_SC_ values from the EQE spectra of the PM6:PY-IT (BC), PM6/PY-IT (SqP), and PM6/PY-IT (SqP-DTA) were 22.71, 22.18, and 22.04 mA·cm^−2^, respectively. The errors of *J*_SC_ values between those calculated from the EQE and those measured from the *J*-*V* measurement were within 5%.

**Figure 3:**
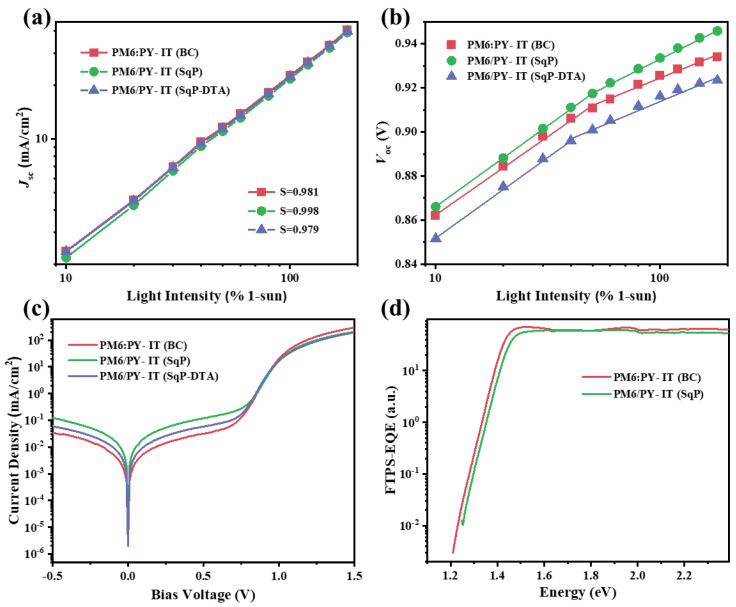
(**a**) *J*_SC_ versus light intensity, (**b**) *V*_OC_ versus light intensity, (**c**) dark *J*-*V* curves, and (**d**) FTPS-EQE spectra measured from 500 to 1800 nm.

To understand the differences of charge recombination among devices based on different active layers, we first performed the dependence of *J*_SC_ on the light intensity (*P*_light_). Figure 3a exhibits the *J*_SC_ and light intensity (*J*_SC_-*P*_light_) curves, which are plotted on a logarithmic scale with a slope of *S,* as listed in Table 1. The closer the parameter *S* is to 1, the weaker the bimolecular recombination in the active layer. The bimolecular recombination of PM6/PY-IT (SqP-DTA) (S ≈ 0.979) was the highest, followed by PM6:PY-IT (BC) (S ≈ 0.981), and PM6/PY-IT (SqP) (S ≈ 0.998) with the lowest bimolecular recombination. This indicates that the SqP method can reduce the bimolecular recombination of the active layer when compared to BC, which is consistent with the trend of FFs of the corresponding devices.

To examine trap-assisted recombination, ideality factors (*n*_id_) were calculated. When the ideality factor is approaching 1, the trap-assisted recombination is less, based on diode theory. Otherwise, more trap-assisted recombination makes the value become higher (typically <2). We can obtain the ideality factors in the following two ways. The first is to calculate ideality factors (*n*_id,l_) under different light intensities by $n_{\mathrm{id},l}=\frac{q}{kT}\frac{\partial V_{oc}}{\partial \ln\left(I\right)}\;$, where *I* is the light intensity. The results are shown in Figure 3b and Table 1. The *n*_id,l_ values of PM6:PY-IT (BC), PM6/PY-IT (SqP), and PM6/PY-IT (SqP-DTA) were 1.248, 1.254, and 1.272, respectively. The *n*_id,l_ values of PM6:PY-IT (BC) and PM6/PY-IT (SqP) were very close, lower than that of PM6/PY-IT (SqP-DTA), which indicates less trap-assisted recombination across the active layer of the SqP-based or BC-based device than that of the SqP-based device with extra thermal annealing (DTA). The second way is to fit the exponential region of the dark *J*-*V* curve, where the dark ideality factor (*n*_id,d_) can be calculated by $n_{\mathrm{id},d}=\frac{q}{kT}\frac{\partial V}{\partial J}$, where *q*, *k*, *T*, *V*, and *J* represent the fundamental charge, the Boltzmann constant, temperature, bias voltage, and dark current density, respectively. The dark *J*-*V* curves are plotted in Figure 3c and the fitting results are listed in Table 1, where *n*_id,d_ values are listed alongside the series (*R*_s_) and shunt resistance (*R*_sh_) of the devices. The *n*_id,d_ values of PM6:PY-IT (BC), PM6/PY-IT (SqP), and PM6/PY-IT (SqP-DTA) were 1.474, 1.449, and 1.496, respectively. The trend of *n*_id,d_ of these three devices was almost consistent with that of *n*_id,l_, with SqP-DTA showing the highest ideality factor and thus the most trap-assisted recombination. These results imply that the SqP method without extra annealing can reduce not only bimolecular recombination, but also trap-assisted recombination, compared to BC.

The high-sensitivity EQE measurements were used here for probing trap states. The sub-gap EQEs were measured using Fourier-transform photocurrent spectroscopy (FTPS), and the spectra of the BC- and SqP-based devices are shown in Figure 3d. It shows clear quantum efficiencies for PM6:PY-IT (BC) in the low-energy region of 1.2–1.25 eV, which is probably attributed to the existence of deep trap states within the bandgap. This may contribute to the relatively poor FF of the device.

**Figure 4:**
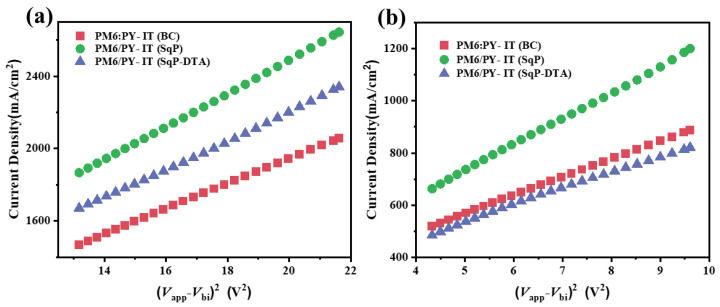
*J*-*V*^2^ curves of hole-only devices (**a**) and electron-only devices (**b**) for SCLC mobility calculation.

To deeply understand the relationship between charge transport property, vertical composite distribution, and device performance, the charge carrier mobilities of active layers processed by different methods were first measured by fitting the dark *J-V*^2^ data of single-carrier devices to the space-charge limited current (SCLC) model. The results are presented in Figure 4 and Table 1. For PM6:PY-IT (BC), unbalanced mobilities for both the hole and electron were measured to be 3.5 × 10^−4^ and 2.3 × 10^−4^ cm^2^ V^−1^ s^−1^, respectively, which is consistent with its inferior photovoltaic performance since unbalanced carriers’ mobilities may result in severe bimolecular recombination to restrain the FF. PM6/PY-IT (SqP) with balanced mobilities for the hole and electron were tested to be 4.1 × 10^−4^ and 4.2 × 10^−4^ cm^2^ V^−1^ s^−1^, respectively, indicating that the SqP method may enhance the PM6/PY-IT phase purity to improve the carriers’ (hole and electron) transport pathway. For PM6/PY-IT (SqP-DTA), the obviously unbalanced mobilities for the hole and electron were 5.8 × 10^−4^ and 2.2 × 10^−4^ cm^2^ V^−1^ s^−1^, respectively, suggesting that extra thermal annealing for the underlayer donor PM6 (DTA) is likely to increase its crystallinity, which is favorable for hole transport, but it restrains the interpenetration between the acceptor and the donor, and finally leads to inferior photovoltaic performance.

**Figure 5:**
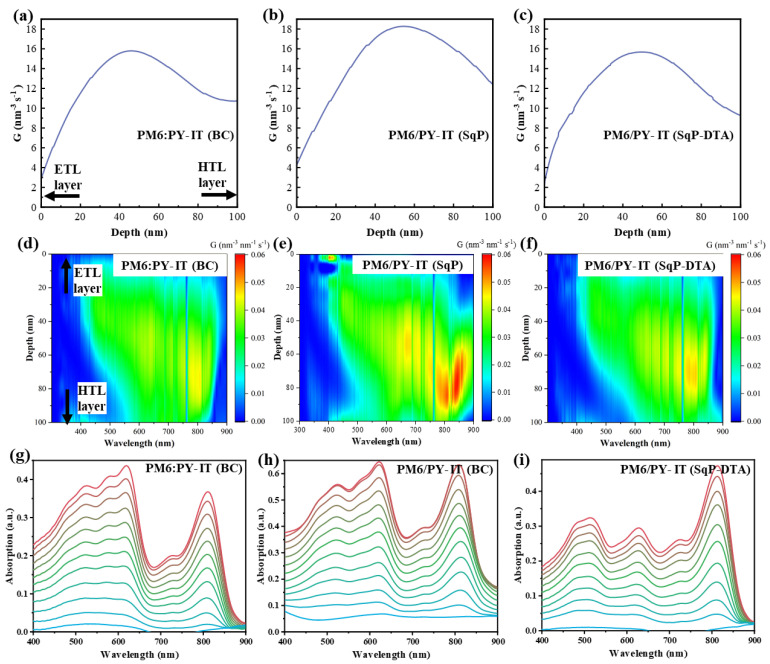
(**a**–**c**) Integrated generation rate in the vertical direction of the film. (**d**–**f**) Exciton generation map across the vertical direction of the active layer film as a function of wavelength. (**g**–**i**) Film-depth-dependent light absorption spectra.

The vertical phase distributions of the donor and the acceptor and exciton generation across the thin film were explored through film-depth-dependent light absorption spectra (FLAS). The active layers were skimmed gradually by low-pressure plasma in a vacuum by precisely controlling the strength of the plasmas and the treatment time. Details are provided in the Experimental Section. Based on the absorption of the sublayers from the top to the bottom of an active layer, the vertical phase separation can be obtained as long as the absorption of the donor and acceptor can be distinguished. The film-depth-dependence light absorption spectra of PM6:PY-IT (BC) and PM6/PY-IT (SqP) have a thickness of 100 nm. The PM6 neat film possesses a strong absorption band at the long-wavelength region peak at 616 nm, while PY-IT strongly absorbs light at the short-wavelength region peak at 825 nm. The results are depicted in Figure 5g–i.

Figure 5a–c show the integrated generation rates for each film in the depth direction. The generation rates were on average higher in the bottom half of the film because the electrons generated need to transport through a longer distance to reach the cathode compared to the holes reaching the anode. The generation rate of PM6/PY-IT (SqP) was the highest among the corresponding devices, as seen from Figure 5a–c, which further emphasizes the importance of electron mobility enhancement by the SqP method.

The energy distributions of photons absorbed in a specific point per unit time in the active layer were calculated by E, and the result of exciton generation rate is shown in Figure 5d–f. It is clearly shown that the exciton generation rate was higher in the bottom half of the device and many more excitons were generated by the SqP method over the BC method, indicating that it is possible to generate larger *J*_SC_ in the active layer using the SqP method.

**Figure 6:**
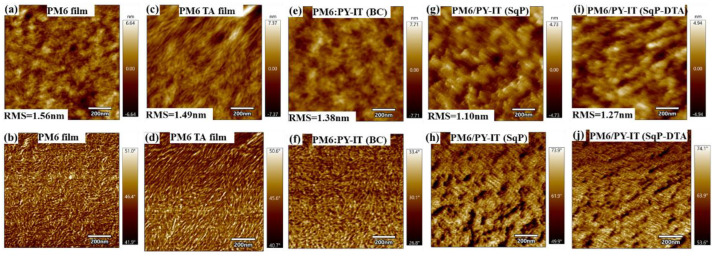
AFM height (top) and phase images (bottom) of (**a**,**b**) PM6 film, (**c**,**d**) thermally annealed PM6 film, (**e**,**f**) PM6:PY-IT (BC), (**g**,**h**) PM6/PY-IT (SqP), and (**i**,**j**) PM6/PY-IT (SqP-DTA).

We prepared pure PM6 films and PM6:PY-IT blend films according to the same procedure used to fabricated devices. For DTA films, we took AFM images on the PM6 film before and after the extra thermal annealing step, and on the SqP blend films as well. The figure below is the new Figure 6 in the revised manuscript. There are several discussion points we can make from the AFM measurements: (1) For pure PM6 films before (Figure 6a) and after thermal annealing (Figure 6c), the roughness was reduced much more after thermal annealing (TA), and TA made the fibrillar structure of PM6 more obvious with elongated fibril chains, suggesting that the crystallinity was enhanced. This also implies that its swelling capability was reduced during the spin-coating of the acceptor’s solution, which is not conducive to the interpenetration of the donor and the acceptor. (2) For both PM6:PY-IT (BC) and PM6/PY-IT (SqP), PY-IT appeared on the top surface. However, compared to PM6:PY-IT (BC), there seemed to be much more PY-IT distributed on the surface of PM6/PY-IT (SqP), which is a more favorable state in terms of the vertical phase segregation of the active layer. (3) The comparison between the SqP and SqP-DTA films indicated that the surface of PM6/PY-IT (SqP-DTA) had less PM6 than that of PM6/PY-IT (SqP), suggesting that excessive phase separation may occur in the active layer, particularly in the vertical direction due to the insufficient swelling for the PM6 underlayer, which is not conducive to the device performance. (4) Compared with SqP devices, the surface roughness of BC devices was higher, which may be caused by the relatively more uniform mixing between PM6 and PY-IT that led to more PM6 in the upper layer than in SqP devices.

## 4. Conclusions

In conclusion, the two-step sequential processing has been successfully employed in the fabrication of all-PSCs based on a polymeric donor (PY-IT) and a polymeric acceptor (PM6), and it achieved PCEs of all-PSCs which were negligibly lower (15.15%) than the corresponding BC-based all-PSCs. Furthermore, by systematically comparing the similarities and differences between the devices prepared by the SqP method and the BC method with respect to their optical and electronic properties, together with the above-mentioned variation in BHJ morphology, we found that SqP presented greatly improved FF, reduced bimolecular recombination, and enhanced phase purity to increase the carrier mobility. The results imply that the SqP method is an effective approach to preciously control the morphology of photovoltaic materials and to fabricate high-performance all-PSCs, especially to realize industrial mass-production in the near future.

## Figures and Tables

**Table 1:** Summary of photovoltaic parameters for PM6 and PY-IT-based all-PSCs processed from different conditions, measured under AM 1.5 G illumination at 100 mW cm^−2^.

Active Layer	*V*_OC_ (V)	*J*_SC_ (mA/cm^2^)	FF	PCE ^(a)^ (%)	*n*_id,d_^(b)^	*n*_id,l_^(c)^	S ^(d)^	*R*_s_^(e)^	*R*_sh_^(e)^	*μ*_h_/*μ*_e_ ^(f)^	Ratio ^(g)^
PM6:PY-IT (BC)	0.934 ± 0.005 (0.937)	23.78 ± 0.25 (23.90)	0.654 ± 0.021 (0.677)	14.49 ± 0.59 (15.15)	1.474	1.248	0.981	1.1	1.0	3.5/2.3	1.54
PM6/PY-IT (SqP)	0.936 ± 0.002 (0.936)	22.90 ± 0.26 (23.19)	0.685 ± 0.011 (0.691)	14.70 ± 0.28 (15.00)	1.449	1.254	0.998	1.1	1.3	4.1/4.2	0.98
PM6/PY-IT (SqP-DTA)	0.930 ± 0.003 (0.932)	22.45 ± 0.45 (22.36)	0.644 ± 0.014 (0.657)	13.44 ± 0.16 (13.69)	1.496	1.272	0.979	1.8	1.0	5.8/2.2	2.28

^(a)^ The standard deviations are based on measurements of over at least ten independent devices. ^(b)^ Ideality factors obtained from fitting dark *J*-*V* curves. ^(c)^ Ideality factors obtained from analyzing *V*_OC_-light intensity data at low light intensities (≤1-sun). ^(d)^ The slope from the linear fit of *J*_SC_ versus log*I*. ^(e)^ The units of *R*_s_ and *R*_sh_ are ohm/cm^2^ and ×10^4^ ohm/cm^2^, respectively. ^(f)^ The units of *μ*_h_ and *μ*_e_ are both ×10^−4^ cm^2^ V^−1^ s^−1^. ^(g)^ Ratio of hole mobility and electron mobility.

## Data Availability

Not applicable.
